# AN INTERNATIONAL COLLABORATION TO SUPPORT AGING RESEARCH IN GHANA

**DOI:** 10.1093/geroni/igae098.1874

**Published:** 2024-12-31

**Authors:** Tiffany Washington, Dennis Bortey, Fayron Epps, Janelle Gore, Venance Dey

**Affiliations:** University of Georgia, Knoxville, Tennessee, United States; Alzheimer’s Ghana, Tema, Greater Accra, Ghana; The University of Texas Health San Antonio, San Antonio, Texas, United States; University of Alabama, Alabama, United States; Alzheimer’s Ghana, Tema, Greater Accra, Ghana

## Abstract

Nearly 3 million people live with dementia in sub-Saharan Africa (SSA). However, ineffective methods for increasing detection and raising awareness contribute to stigma about dementia. By leveraging the resources of universities in developed countries, global community engaged research can increase knowledge and education about dementia in SSA. Using a collaboration between Ghanian community partners and two research-intensive universities in the U.S. as a case example, this paper offers guidance on conducting community-engaged aging research in Ghana. The Alter Program is a dementia education initiative to increase dementia-friendly spaces in faith communities. We used thematic analysis of field notes to identify key components of building international partnerships centered on dementia education. Five were identified: ethics review, relationship-building with community partners, identifying community needs, cultural adaptations, and capacity building. Future research can leverage these aspects of community-universities partnerships to increase dementia knowledge and attitudes in SSA countries.

